# Bell-X, An Opportunistic Time Synchronization Mechanism for Scheduled Wireless Sensor Networks

**DOI:** 10.3390/s19194128

**Published:** 2019-09-24

**Authors:** Jose Vera-Pérez, David Todolí-Ferrandis, Javier Silvestre-Blanes, Víctor Sempere-Payá

**Affiliations:** 1 Instituto Tecnológico de Informática, 46022 Valencia (ITI), Spain; dtodoli@iti.es; 2 ITI and Universitat Politècnica de València (UPV), DISCA, 03801 Alcoy, Spain; jsilves@disca.upv.es; 3 ITI and Universitat Politècnica de València (UPV), DCOM, 46022 Valencia, Spain; vsempere@dcom.upv.es

**Keywords:** WSN, synchronization, IoT, IIoT, TSCH

## Abstract

The Industrial Internet of Things (IIoT) is having an ever greater impact on industrial processes and the manufacturing sector, due the capabilities of massive data collection and interoperability with plant processes, key elements that are focused on the implementation of Industry 4.0. Wireless Sensor Networks (WSN) are one of the enabling technologies of the IIoT, due its self-configuration and self-repair capabilities to deploy ad-hoc networks. High levels of robustness and reliability, which are necessary in industrial environments, can be achieved by using the Time-Slotted Channel Hopping (TSCH) medium access the mechanism of the IEEE 802.15.4e protocol, penalizing other features, such as network connection and formation times, given that a new node does not know, a priori, the scheduling used by the network. This article proposes a new beacon advertising approach for a fast synchronization for networks under the TSCH-Medium Access Control (MAC) layer and Routing Protocol for Low-Power and Lossy Networks (RPL). This new method makes it possible to speed up the connection times of new nodes in an opportunistic way, while reducing the consumption and advertising traffic generated by the network.

## 1. Introduction

The increasing popularity of applications that are derived from the concept of Industry 4.0 is leading to the emergence of new requirements in the scope of connectivity and data collection. Giving support to this need for massive data and flows of information, interconnecting everything and enabling the digital transformation, is the role of technologies labelled under the Industrial Internet of Things denomination (IIoT). Among these technologies, a favourite candidate is the Wireless Sensor Network (WSN) [1], because it provides a faster, more flexible, scalable, and cheaper solution to deploy than its wired counter-part.

Nevertheless, the adoption of WSNs, especially for industrial applications, raises several challenges that need to be addressed before considering it a mature solution that is capable of hitting the market successfully. Any network deployed to operate in an industrial setting requires a high degree of reliability, which translates into enhanced connection times and avoidance of downtimes due to power or traffic loss.

Traditionally, wireless communications have not been widely adopted or popular in industrial scenarios due to the harsh environment and its impact on these type of communications. Radio interferences or signal fading due to multi-path propagation and metal and other material obstacles are common issues that hinder transmissions, which, actually, must be received with the according reliable delivery QoS (Quality of Service), and not with best-effort service behaviour.

In response to these requirements, in the scope of WSN and IEEE 802.15.4 protocol [2] (a standard for low-power wireless personal area networks that operate in short to middle range, with distances up to 100m per hop in mesh networks), the IEEE802.15.4e amendment (first approved in 2012) defines a group of different medium access control modes. Among the described modes, the Time Slotted Channel Hopping (TSCH) [3] stands out as a solution that enables frequency channel jumps to palliate interferences.

These TSCH based networks, when combining a limited number of nodes with deterministic time behaviour that helps to limit latency and lost traffic, show promising results to be a suitable candidate for manufacturing instrumentation and monitoring tasks [4], which, using IEC 62264 (ANSI/ISA 95) nomenclature, can be defined as:

- Flow based process monitoring (latency 100ms. loss probability below 10%–6%, and number of devices below 30) and Supervisory control (latency 10s. loss probability below 10%–6%, and number of devices below 100).
- Job based factory monitoring (latency 100ms. loss probability below 10%–7%, and number of devices below 10).

On the other hand, the IPv6 Routing Protocol for Low-Power and Lossy Networks, known as RPL [5], is one of the most adopted solution in WSNs, and it can used over IEEE802.15.4e or even other link layer options to achieve a robust deployment with networking capabilities, mesh and multi-path support, and integration and interoperability with IP networking.

Nevertheless, this deterministic behaviour has certain drawbacks during the initial deployment stages and recurrent situations during the whole network time operation. When there is a lack of synchronization between the joining node and the rest of the network, successfully connecting (i.e. not only synchronizing clocks, but also joining the network effectively) can be challenging. This problem does not only show up during initial deployment, but also after replacement of batteries, after unexpected disconnections due to external factors, etc. This issue might appear due to several factors that the authors analysed previously in [6], although this study focused on presenting a solution for achieving better connectivity success without compromising energy consumption, for the initial deployment stage.

Derived from the conclusions of said work, an improved and configurable synchronization and connection mechanism, named Bell-X, is presented in the following article, which allows for finding a trade-off between connection time and energy consumption, while achieving 100% connectivity success. Bell-X allows for modifying dynamically the transmission period of the Enhanced Beacons, needed for the synchronization process. This beacon transmission period will be periodically re-configured using different tuneable parameters that modify the performance of the mechanism, enabling different beacon transmission zones. Zones with a lower beacon transmission period allows for the speeding up of the synchronization and connection phases, while a higher transmission period compensates this performance in order to obtain a duty cycle smaller in average and to save energy by reducing advertising traffic.

The rest of the article is structured, as follows. Section 2 introduces a brief description of the TSCH access method and the RPL dynamic routing protocol, in order to understand how the medium access, synchronization, and networking is achieved in these WSNs. Section 3 introduces related work addressing the problem of synchronization and connectivity in WSNs. Section 4 shows the problem statement and the proposed mechanism and advertising method. Section 5 describes the testbed, experiments, and simulations that were used to achieve the results analysed in Section 6. Closing the article, Section 7 offers conclusions and possible future improvements.

## 2. Timing for TSCH and RPL

In the WSN research field, timing is of great importance for accomplishing deterministic communications, since it is necessary to form and maintain a precise synchronization between the different nodes forming the network, in order to manage the generation of messages, so nodes on the receiving end know when to have their radio enabled to detect these messages. The following subsections show an overview of the TSCH and RPL protocols, describing what timing mechanisms are used to generate the necessary advertising and control messages for each protocol. An in-depth explanation of these protocols can be found in [6] and in their respective standard and RFC documents.

### 2.1. TSCH Beacon Advertising

The IEEE 802.15.4 standard defines different medium access mechanisms, highlighting the TSCH mode for those applications that require high reliability, especially for industrial scenarios where interferences are more common. TSCH medium access mode allows for structuring the temporal plane following the philosophy that was inherited from the TDMA protocol, while using a certain number of slots that are periodically repeated in time. This subset of time slots can be grouped into what is known as slotframe. In addition, TSCH allows for different communication channels to be used, using a frequency hopping technique, which allows a different channel to be used during each time slot, in order to mitigate interferences. To be able to calculate the frequency channel used in each time slot, the following equation is used:

(1) $$f=F\left\{\left(ASN+ch_{offset}\right)mod\;C\right\}$$

where f is the physical transmission channel; ASN is the absolute slot number, which corresponds to the number of slots that have passed since the network was deployed; the $ch_{offset}$ parameter allows for different channels to be used in the same time slot; and, C corresponds to the total number of channels that are used. Therefore, the function F{−} consists of selecting a channel of a predefined sequence according to the index that is obtained after calculating the module.

The scheduling of these radio resources (time slots and frequency channels) allows for optimizing the performance of the nodes within the network, enabling only those slots in which a message exchange is scheduled, and leaving the rest inactive to save energy. These rules to establish an optimized scheduling are not defined in the standard, but there are recommendations to establish minimum resources to carry out a basic exchange [7].

In the TSCH mechanism it is necessary to perform a previous synchronization phase in order for a node to be able to access the network. In this phase, the node attempting to join the network must acquire information about the scheduling used, as well as adjust its time clock based on a reference node that already belongs to the network. This synchronization is necessary so that two devices can exchange messages within the same timeslot. During this slot time, a node must be able to send a message and receive its corresponding acknowledgement. The exchange of these messages would not be possible if two nodes are not correctly synchronized.

The nodes that are already correctly synchronized are periodically sending advertising messages with information about the scheduling or the current value of the ASN parameter in order to carry out the synchronization process. These messages are known as Enhanced Beacons (EB), an improved version of traditional IEEE 802.15.4 beacons that allow for additional information to be included in fields called Information Elements.

Moreover, the nodes that were prepared to join the network start the scanning process, during a certain preconfigured period and for each of the configured channels, until they correctly receive an Enhanced Beacon. The synchronization process will take longer depending on different factors such as the number of channels configured, the scan time of each channel, the traffic on the network or the Enhanced Beacon transmission period [6].

**Figure 1:**
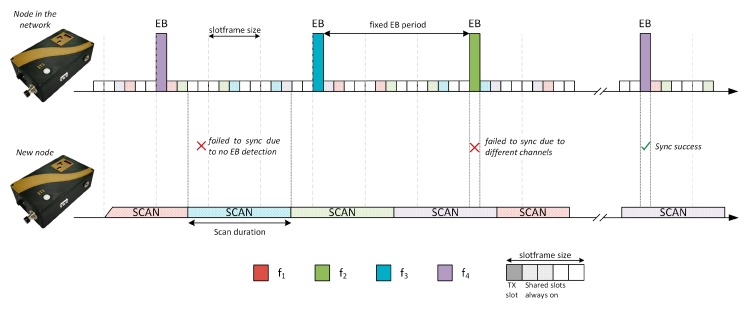
Enhanced Beacon (EB) scan process in Time Slotted Channel Hopping (TSCH) [6].

Figure 1 shows a scheme with two nodes, one synchronized and sending beacons and another during the scanning phase. The scheme illustrates different reasons why the synchronization process might fail. Taking these cases into account, the configuration of parameters, such as slotframe size, beacon advertising period, or scan time, can improve the synchronization times of new nodes.

### 2.2. Active Channel Scan Method

The configuration of the parameters that are involved in the synchronization process is not described in the standard, and its optimization will depend on use case restrictions, such as fast deployment or energy savings. For the scanning phase, the standard proposes different variations that could speed up the process of receiving Enhanced Beacons, an active and a passive mechanism. In the passive scanning mechanism, a node scans in the different channels until it receives a beacon correctly. On the other hand, the active method allows a beacon request to be sent every time the channel is changed, so synchronized nodes can respond to the request with an Enhanced Beacon.

**Figure 2:**
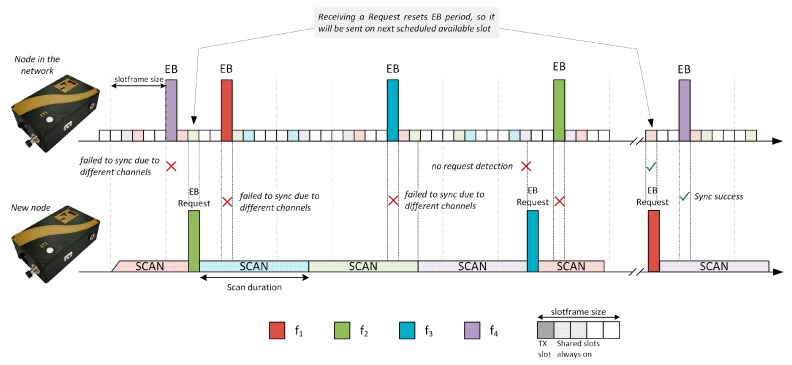
Active scanning method implemented for TSCH.

In order to improve the synchronization process and reduce the connection times for TSCH networks, a brief test has been carried out to validate the viability of an active scan method in TSCH. For these tests, the TSCH implementation in the nodes was modified in order to force the activation of transmitting radio, as the normal behaviour of the Medium Access Control (MAC) layer is to discard any packet in the outbound buffer if the node is not synchronized with a TSCH network. Without bypassing this filter, the nodes were not able to send any request without previously receiving an EB, and therefore rendering the active method useless. The implemented behaviour can be seen in Figure 2.

The test implemented included six nodes: five of them actively scanning and sending requests, and one central node sending EBs, but configured with an infinite period so the new nodes never receive an unrequested (passive) beacon. In this way, counting the number of transmitted and received requests allows for the probability of receiving beacon requests from non-synchronized nodes to be calculated. In view of the results that were obtained, as seen in Table 1 it can be concluded that an active scanning method is not useful in TSCH networks, since the probability that synchronized nodes receive a beacon request is too low.

### 2.3. RPL Trickle Timer

It is necessary to build a network topology once the devices achieve synchronization with the TSCH slotframe of their neighbours, so that the nodes know the best routes to carry out multi-hop communications. Wireless Sensor Networks consist of a set of constrained devices, limited by either computing power, memory, or energy consumption. The RPL protocol calls these types of networks Low-power and Lossy Networks (LLN). RPL is specially designed to be able to establish routing rules in networks with low-resource devices. For this reason, the RPL protocol is widely used as a de facto standard for establishing routes and maintaining the topology in meshed wireless networks. Using a set of objective functions, the RPL protocol allows for the nodes to dynamically and autonomously update their routes, estimating the quality of the links through different metrics.

For the formation of the RPL DODAG (Destination-Oriented Directed Acyclic Graph) tree, different ICMPv6 control messages are used. DODAG Information Object (DIO) messages are sent by the nodes of the RPL network to keep the routes updated and allow for new nodes to join the network. DODAG Information Solicitation (DIS) messages are sent to request that nodes that are already in the network send a DIO message with the RPL information that is needed to join the topology. Finally, Destination Advertisement Object (DAO) messages allow for the ascending routes to the coordinator node of the RPL tree to be created.

**Figure 3:**
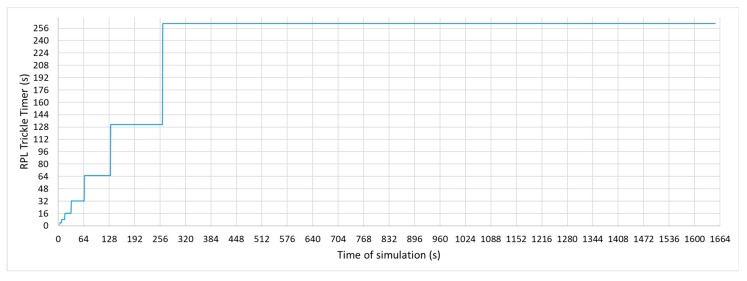
Routing Protocol for Low-Power and Lossy Networks (RPL) Trickle Timer evolution over time.

RPL protocol uses a Trickle-based timer, described in RFC6206 [8], to carry out the dissemination of DIO messages in such a way that control traffic is significantly reduced when the RPL topology is stable. This algorithm allows for the DIO Trickle timer value to be modified as long as the information received does not indicate that the topology has undergone any changes. The DIO Trickle timer will be reset when an inconsistency in the topology is detected or when a multicast DIS is received. In Figure 3, the evolution of the RPL Trickle Timer is shown. Each time the timer expires, the algorithm doubles its value until it reaches its maximum value. In the case shown in the graph, the Trickle Timer reaches its maximum value in a few minutes.

**Figure 4:**
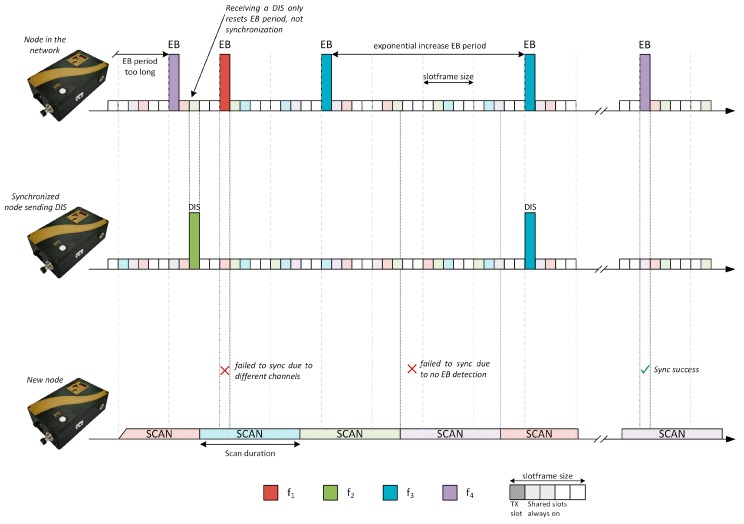
Enhanced Beacon transmission period triggered by RPL Trickle Timer [6].

In the real implementations of a communication stack, such as the one carried out by the Contiki OS [9], the TSCH medium access mechanism uses the RPL Trickle timer to modify the EB transmission period. This modification allows for advertising traffic to be reduced, as is the case with control DIO messages. However, this change is controlled by RPL, where the DIO messages used to keep the routes updated do not have the same criticality as the messages to keep the network synchronized. Figure 4 shows a representation of the beacon transmission mechanism, modified with the RPL trickle timer.

## 3. Related Work

As the TSCH mechanism was included in the 2012 amendment of the IEEE 802.15.4 standard, different proposals have been conducted to improve different aspects of the aforementioned protocol. There are certain procedures that are not fully described in the IEEE 802.15.4 standard, either because there are different solutions that fit for different domain applications or because the optimization of different procedures are outside the scope of the standard. In this way, only those mechanisms that allow for TSCH to function properly, even if not optimized, are defined in the standard. The scheduling of the TSCH radio resources, in time and frequency, and the advertising message generation for time synchronization are some examples that have been studied in recent years.

It is important to keep a minimum error between the drift clocks of different devices in order to maintain a precise synchronization between two devices. That is why different studies have been carried out [10,11,12,13], evaluating the main problems that a poor estimation of the drift clock may cause. These authors propose an adaptive synchronization mechanism, which makes it possible to improve the estimates of drift clock in multi-hop networks. These implementations are widespread and they can be found integrated into WSN development environments, such as Contiki OS.

However, although the nodes are able to maintain a very precise synchronization once they are connected, the pre-synchronization process is a problem widely analysed in the literature in recent years. The articles [14,15] perform different analyses showing the problems and open fields of study on the TSCH mechanism, pointing out that the synchronization and maintenance of the network is still a subject of study. Different approaches have been adopted in the literature, such as enabling a dedicated scheduling to signalling traffic, increasing the number of nodes that send beacons, reducing the probability of collision by assigning guaranteed slots, using fewer channels in the scanning process or modifying the beacon transmission period to improve the synchronization process in TSCH networks.

The authors in [16,17,18,19,20] propose different schemes for the TSCH scheduling to favour the connection process, enabling different slots for the signalling plane. Vogli et al. [16] propose two different schemes to speed up joining operations, allowing for the nodes to use a random channel or an advertising slot to send periodically EB frames. Duy et al. [17] propose a different scheme in which the nodes can dynamically determine the number of EB needed for the synchronization. Khoufi et al. [18] compare with the state of the art a new scheme that allows for beacons to be sent through all channels of the TSCH network without collision, improving the results proposed in [16]. Authors in [19] define another scheme to allow for the nodes to estimate the best channel to send EB, oriented toward being more efficient in noisy environments. Karalis et al. [20] propose a non-centralized EB scheduling that is free of collisions, even considering mobile nodes.

All of the aforementioned solutions need to allocate more time slots in the scheduling of TSCH, which leads to greater energy consumption even when no message is transmitted. Karalis [21] propose a different technique that allows the EB rate to be increased without the need to allocate more timeslots, sending multiple EBs in different channels in a single timeslot.

A performance analysis is carried out by the authors in [22], which shows a synchronization mechanism that randomly sends EB messages. They also analyse the influence of other parameters such as the number of channels involved in the synchronization process. Wang et al. [23] perform different simulations, in which the density of nodes and the transmission beacon period varies, showing that a random period improves the connection process.

Vallati et al. [24] analyse, when considering the entire protocol stack, the problems of the synchronization process in TSCH networks, comparing the minimal configuration proposed by 6TiSCH group and their proposed dynamic resource management algorithm. In [25], a dynamic EB period is proposed, sending more advertising messages, based on the number of joined nodes.

In [6], the authors introduced a mechanism that was developed to facilitate the deployment of a WSN network, addressing possible issues during synchronization and connection phases when joining the network, and the influence of the TSCH and RPL parameters. This work proposes a mechanism that improves connection time and success probability, while significantly reducing energy consumption. This is achieved by allowing for the Beacons transmission period to be increased once the initial network set-up is finished and the network is in stable condition. This proposal is based on the analysis of results that were obtained by the methods described as “Fixed EB” and “RPL trickle timer”. Using a Fixed EB, to achieve adequate synchronization and connection times, low periods must be used, with the consequent increase in energy consumption. Using the “RPL trickle timer”, as in the proposal used in Contiki, poor synchronization and connection results are obtained, since this mechanism, valid for CSMA-CA (Carrier sense multiple access collision avoidance) RPL networks, does not take the particularities of TSCH networks into account. To address this, the Custom Trickle Timer was developed in [6], which allowed for the high synchronization and connection probabilities of using a fixed and small EB transmission period to be matched with a low energy consumption, similar to that used with high periods (penalized with very poor connection results).

The designed mechanism is valid during the set-up of a network, but did not address the problem of reconnection or connection of new nodes to the network after a long period of operation. A possible solution contemplates the use of an active mechanism, such as the one based on the sending of DIS messages to restart the timers. However, these RPL messages are discarded by the TSCH layer, as they are not yet synchronized. In this paper, the software of these layers was modified to allow for the real transmission of these messages. However, the chances of success of receiving this message before synchronizing is 1%–2%, as seen for the active scanning method, which implies a low impact in successfully joining the network, but with high impact in energy cost. Therefore, to achieve an adequate synchronization and connection rate, it is necessary to develop a mechanism that, depending on the scenario, determines the highest acceptable time for synchronization and connection, the lowest possible energy consumption, and modifies the behaviour and parameters of the network, to ensure these results.

## 4. Problem Statement and Proposed Advertising Method

The TSCH medium access method has proven to be one of the most relevant solutions that are included within the IEEE 802.15.4 standard, thanks to the fact that it is aimed at providing the systems with a deterministic behaviour that guarantees a certain degree of reliability and robustness, which are essential characteristics in industrial settings. For this reason, many proprietary communications standards for wireless sensor networks rely on this same dynamic [26]. Although it is true that once the nodes are synchronized TSCH provides great reliability to communications, the mechanism has certain difficulties when it comes to forming the network in a fast and energy efficient way, as mentioned in related works.

The RPL protocol is also an indispensable tool in the formation of sensor networks, providing connectivity and routing capabilities to the devices once synchronization has been acquired. These two protocols were not originally designed to work with each other, and some procedures, such as sending DIS, are relegated to the background when working with a deterministic scheduling, such as that used in TSCH. With the aim of improving the synchronization phase, these two protocols have also combined some of their characteristics. In Contiki OS, it is possible to configure the EB transmission period so that it follows the same evolution as the timer used in the Trickle Timer of RPL, obtaining the same behavior when transmitting beacons than when sending RPL control messages. Although this combination can help to reduce traffic and consumption, the value of the Trickle Timer evolves so quickly that it reaches its maximum value in a few minutes, thus hindering the synchronization process of the nodes if it is not carried out during the first instance of the deployment.

A dynamic beacon transmission mechanism was proposed during the work done in [6]. This mechanism was focused on improving the synchronization process during the initial deployment phases of the network, setting up a beacon22 transmission period low enough for the nodes to connect quickly, then changing to a much smaller advertising traffic. This fast connection allowed to save time and energy, since during the scanning phases the duty cycle of the radio is close to 100%, so long scanning times can significantly affect the battery level of the devices. In addition, if a node synchronizes quickly during the deployment phases, the Trickle Timer will not yet have reached its maximum value, so the connection with the RPL topology will also be faster.

The conclusions of this work showed that a dynamic mechanism for network set-up could improve the work of deploying a network if we compare it with both configurations, the fixed transmission period and the period modified by the Trickle Timer, since messages that are oriented to synchronization in TSCH have different requirements than the RPL control messages. However, there are situations in which new nodes are added to the network when it is in a stable state, such as restarted nodes, replacement of devices or batteries, or simply an expansion of the network in which several nodes are added to an existing network. This flexibility is a main feature that should be highlighted in wireless networks and the main reason to design a new mechanism that addresses these network needs. Moreover, it is of special interest after assessing the poor results of active scan mechanisms in these networks. In the next section, the proposed operation of a dynamic and configurable EB transmission mechanism is presented.

### 4.1. Dynamic and Configurable Transmission Period Mechanism

**Figure 5:**
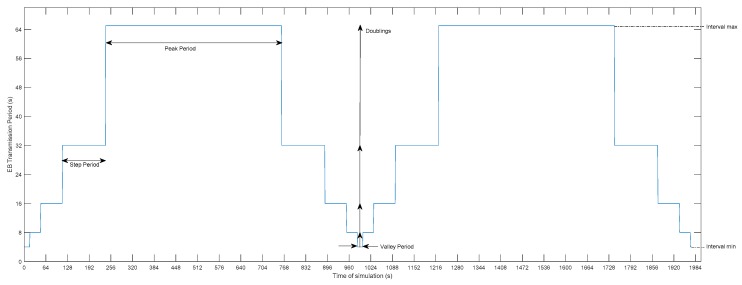
Example of a possible Bell-X configuration (Interval min: 4 s—Doublings: 4—Valley factor: 4—Step factor: 4—Peak factor: 8).

The proposed mechanism for the transmission of EB messages consists of a dynamic and configurable timer, whose temporal response resembles a stepped bell that is periodically repeated in time. Derived from the shape of the temporal representation of this beaconing timer, the mechanism is called Bell-X (with the X representing the tuneable parameter that defines the timer behaviour). In this mechanism, peak zones can be appreciated, in which the beacon transmission period is set to its maximum value, so that the consumption due to the transmission of advertising messages will be minimized. On the other hand, the timer will periodically go into valley areas to send more beacons during a certain time that can be preconfigured. Depending on the requirements of the application domain, there will be a compromise relationship between connection time and energy consumed. During this time in which the timer is reduced, a window is created in which the connection success probability of new nodes is favoured, while the peak areas allow for compensating the energy consumption that the sending of more messages requires. The behaviour of EB messages sent during the valley period could resemble the sending of message bursts to expedite the synchronization process. Between the zone of peak and valley, the transition is carried out in a staggered way, so that the beacon transmission period is doubling each time the timer set in each step expires, in the same way that the RPL Trickle Timer behaves. Figure 5 shows the temporal evolution of one possible configuration of the proposed mechanism, indicating the periods of valley, peak, and intermediate steps.

As already mentioned above, this mechanism allows for configuring certain aspects of the bell’s shape, so that desired behaviors can be configured depending on each type of application. Table 2 shows a list of the configuration parameters.

The description of the operation of the proposed mechanism is as follows:

1. Once the nodes are synchronized and connected to the topology, the dynamic mechanism starts the beacon transmission period by setting it to the value indicated by the Interval min parameter.
2. In addition to setting the beacon period, a timer is started with a duration equal to the value of the Interval min parameter multiplied by the value of the Valley Factor parameter. At this point, the beacon transmission period is set to its minimum value, allowing as many beacons as indicated in the Valley Factor parameter during the valley zone to be sent.
3. Once the configured timer has expired, the dynamic mechanism modifies the transmission beacon period by doubling the previous value of the beacon period. Again, a new timer is configured while using the current value of the beacon period and the Step Factor parameter. This same process will be repeated as many times as the value of the beacon period is doubled.
4. When the beacon period is set to its maximum value, the timer used will be the current value of the beacon period multiplied by the Peak Factor parameter. The maximum value of the beacon period must be enough to maintain synchronization with the nodes that already belong to the network. This work [12] analyses the drift clock estimation that was used in TSCH networks, proposing an adaptive synchronization that obtains drifts estimation lower than 10 ppm in multi-hop networks. This allows configuration of transmission periods with values between 50 and 130 s, depending on the amplitude of this drift.
5. Once the time in which the beacon period is at its maximum has passed, the process will be reversed, which reduces the value of the beacon period until it reaches the valley area. This procedure will be repeated periodically over time, as can be seen in Figure 5.

By adjusting the values of the three parameters controlling the period in each zone, it is possible to control the duty cycle of the signaling messages and the impact on energy consumption that such a configuration will have.

### 4.2. Reset Triggered by Higher Layers of the Protocol Stack

**Figure 6:**
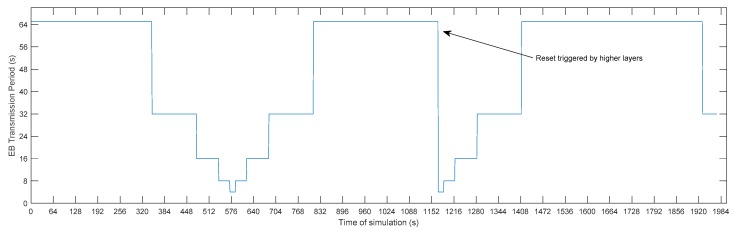
Evolution of the beacon period and reset triggered by higher layers.

A procedure by which it is possible to reset the status of the current bell has been included in order to allow for the mechanism to act against events that may occur in the network. In this way, it is possible to reset the value of the beacon period when there is a change of RPL parent or even through a user-level request. The mechanism can be restarted when it receives any request from the upper layers of the protocol stack. Figure 6 shows the evolution of the beacon transmission period in time, highlighting the moment in which the normal process of the bell is interrupted by a restart that is triggered by the upper levels of the stack.

### 4.3. Dynamic Configuration Depending on the Role of the Node

One of the advantages of the proposed dynamic mechanism is that each of the nodes in the network can have a different configuration, regardless of the rest of the nodes. In addition, even if the nodes have the same bell configuration, the timers do not start synchronously, so that the representation of the bells will be temporarily shifted from each other. As the configurations are independent, it is possible to configure different bells depending on the role that the node has within the network, using a bell with a higher duty cycle if there are routing nodes that are powered, and a more energy efficient bell for those nodes using batteries.

**Figure 7:**
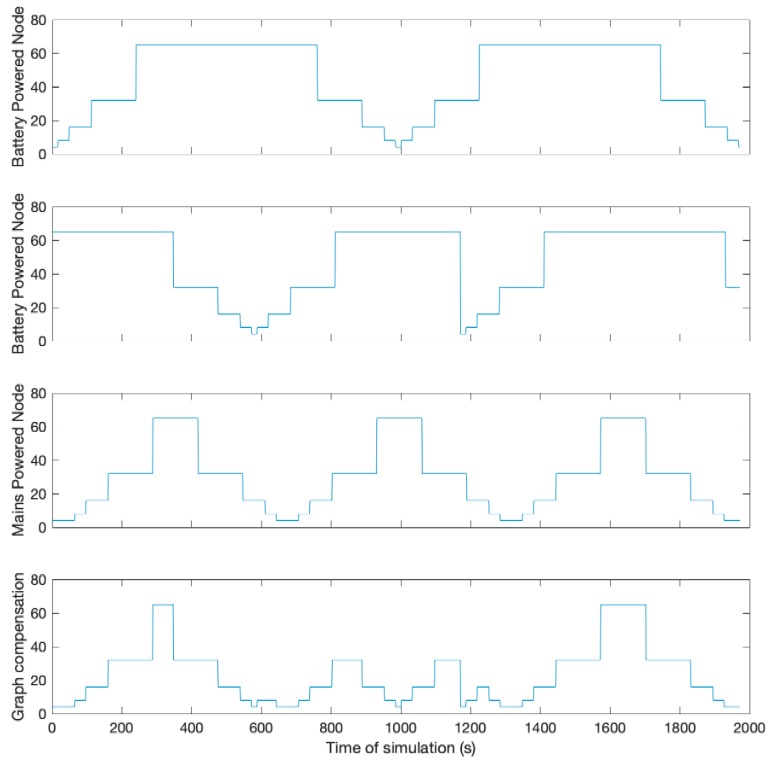
Compensation between nodes of the beacon transmission mechanism.

In Figure 7, the temporal evolution of the beacon transmission period for three different nodes is shown, two of them with a long peak period, oriented to nodes with batteries, and one of them with a more extensive valley area, designed for mains powered nodes. The bottom chart shows a combination of the other three devices, so that the valley zones of other nodes can compensate the peak zones if a new node has three neighbours with different configurations.

## 5. Simulations and Testbeds

Different simulations have been carried out to achieve the performance analysis in comparison with the state of the art beacon advertising methods in order to evaluate the proposal method. To be able to assess the viability of a transmission mechanism, it is important to consider different performance aspects, such as the probability of success for both TSCH synchronization and RPL connection, the energy consumption, related to the traffic generated, or the time that a new node takes in obtain the synchronization when compared to other approaches.

All of the different protocol stack variations have been implemented based on the Contiki OS, using TSCH as the proposed medium access solution. Contiki includes the Cooja simulator, which allows for the behaviour of different types of WSN platforms to be simulated in order to evaluate the Contiki protocol stack.

The result that was obtained in [6] showed that a dynamic mechanism could improve the scanning process during the phases of network deployment, reducing the connection times and improving energy consumption.

For this reason, these simulations are focused on demonstrating the correct functioning of the proposed mechanism during the steady state phase of the network, when the RPL Trickle Timer has reached its maximum value and all of the nodes of the topology maintain a stable connection.

**Figure 8:**
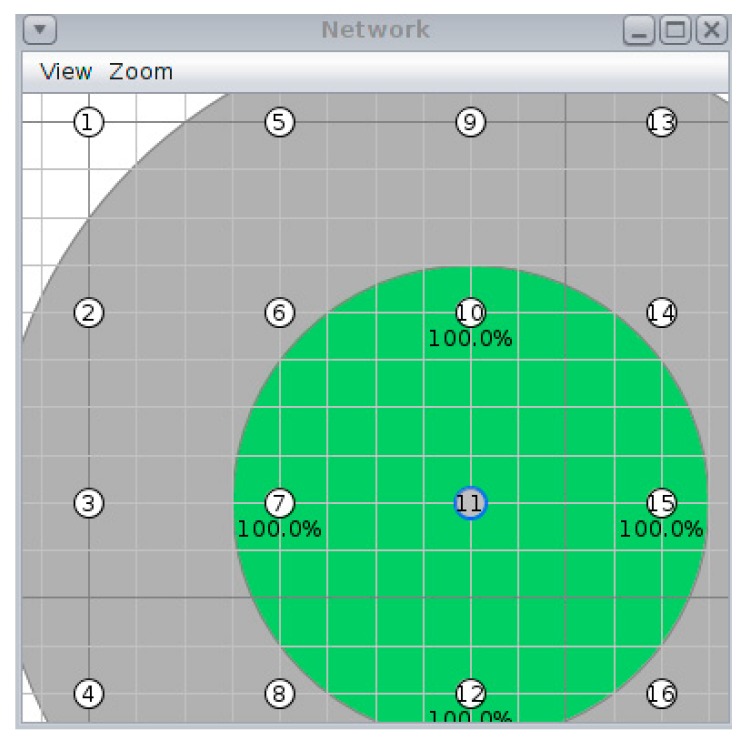
Physical topology for all simulations.

The chosen topology is formed by a meshed network of 16 nodes, in which the upper left node is the coordinator that starts the network. This type of regular topology has been chosen so that the results are not affected by variations in the number of neighbours, since with more nodes transmitting beacons, synchronization is achieved faster. If random topologies were used, the results would be influenced by different external factors that would not allow for the different simulated configurations to be compared on equal terms. Figure 8 shows a capture of the simulated topology in Cooja for each of the configurations under study.

Table 3 summarizes those physical parameters that control the simulation. The nodes are 40 m apart from each other to favour multi-hop communications. For each configuration, 15 simulations have been carried out, with a maximum duration of one hour.

Table 4 shows all of the parameters that are involved in the synchronization and connection process. For the TSCH scheduling, the Orchestra mechanism has been chosen, since it allows for creating a dedicated slotframe for different traffic planes. Regarding the RPL protocol, during the simulations that were carried out in [6], it was observed that the Trickle Timer reached very high values that hindered the process of discovering the RPL topology, for this reason, a Doublings value has been chosen that allows the maximum value of the RPL Trickle timer for each of the configurations to be reduced.

Eight different types of simulations have been carried out, each with a different configuration and repeated 15 times with different seeds, in order to compare the behaviour of the proposed mechanism with the state of the art procedures. Performing a total of 15 repetitions allows for obtaining an average value of the results to be able to compare the different configuration proposed. The simulations consist of two differentiated phases. In the first phase, the 16 nodes of the network must be synchronized and correctly connected. Since this phase corresponds to the initial deployment of the network, a fixed beacon transmission period has been configured during the first four minutes, so that all of the nodes connect quickly, but consume a lot of energy. After these four minutes, the beacon transmission period will function normally depending on the configuration being simulated. This first phase will not be considered in the analysis of results and it is only used to leave the network in a stationary state.

During the second phase, simulations run until minute 20, when all of the nodes have reached the steady state. From this moment on, node 11 will be restarted to observe the time that it takes to get back the synchronization and connect to the RPL topology.

**Figure 9:**
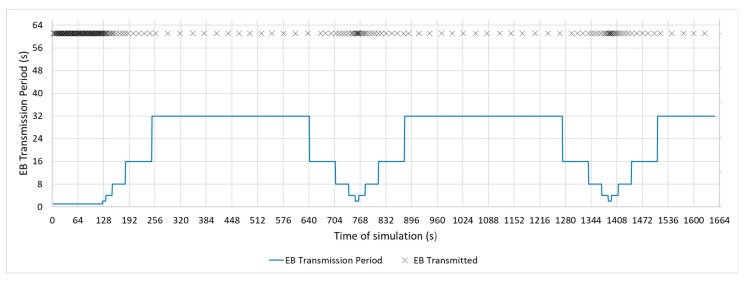
Bell-32 Configuration.

**Figure 10:**
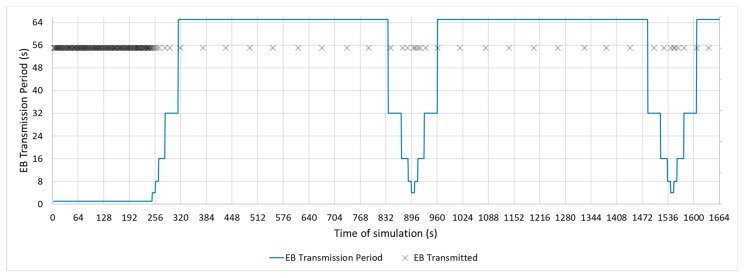
Bell-65 Configuration.

The following figures show the beacon-advertising pattern for every single configuration analysed. Figure 9 and Figure 10 show the proposed Bell-X mechanism with different bell configurations, the first one with an Interval Max parameter of 32 s and the second one with 65 s. From now on, these configurations will be called Bell-32 and Bell-65, respectively. Simulations trace the position in the stepped bell (this is, the value of beaconing period, from valley to peak) of node 11 neighbours so all cases are taken into consideration. In this article, the X of the Bell-X mechanism represents the maximum value that the beacon transmission period can reach, but the rest of the parameters have the same relevance when controlling the Bell’s behaviour. For this reason, it is possible to have different Bell-X configuration under the same name. Table 5 summarizes the configuration parameters for the Bell-X configurations that were proposed.

These two configurations have been chosen to evaluate two different behaviours of the Bell-X mechanism. Bell-32 allows for a faster connection while Bell-65 saves energy by sending less advertising traffic. By configuring the values of the Valley Factor, Step Factor, and Peak Factor, it is possible to control the percentage of time that the transmission period is configured with the maximum value. This means, for energy savings, the selected configuration should include longer Peak periods and higher Interval Max, while faster network connectivity is achieved for longer valley periods and shorter Interval Max.

**Figure 11:**
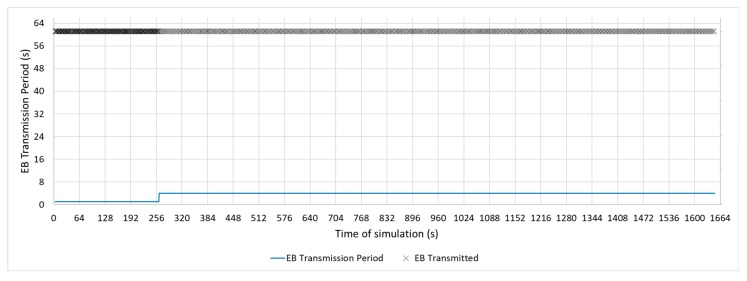
M4s Configuration.

**Figure 12:**
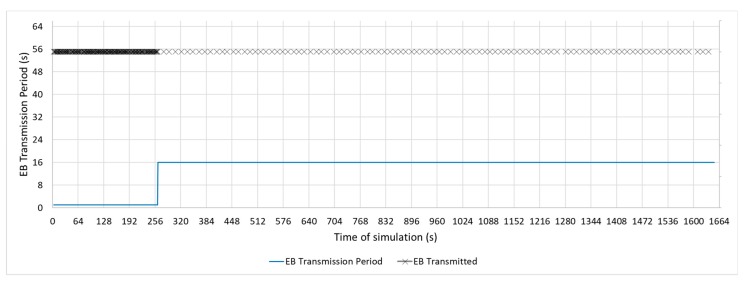
M16s configuration.

**Figure 13:**
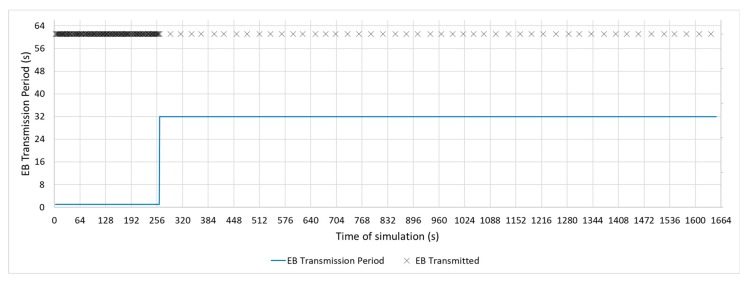
M32s configuration.

**Figure 14:**
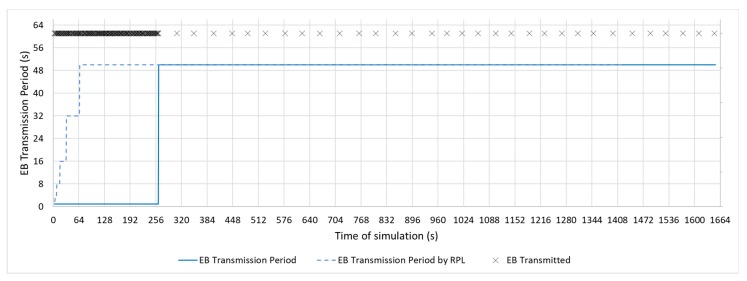
MT configuration.

Figure 11, Figure 12 and Figure 13 show a configuration in which the beacon period is a fixed value of 4, 16, and 32 s, respectively. These three configurations with a fixed periodic advertising fit the solution that is proposed in the standard, which is the basis for the Contiki implementation. From now on, these three configurations will be called M4s, M16s, and M32s. Finally, Figure 14 shows a configuration in which the beacon transmission period is modified by the RPL Trickle Timer. As the Trickle Timer arrives at its maximum value of 50 s before the end of the first phase of the simulation, once phase two begins it is set directly to its maximum value. For this reason, Figure 14 also includes the representation of the Trickle Timer with a dotted line. From now on, this configuration will be called MT. In addition, in each of the figures explained above, the beacons that are sent during a simulation are marked with an ‘x’ pointer.

In Table 6, the consumption that corresponds to the CC2420 radio is shown. These values have been used for energy analysis since in the Cooja simulations the node type Zolertia Z1 has been chosen. The values show the energy consumption for each type of slot possible in the communication.

## 6. Results

The following sections summarize the results that were obtained by the previously described simulations and experiments. The analysis of gathered data focuses on three Key Performance Index (KPIs):

- Connection success rate, because there are configuration cases in which nodes are not always able to join the network.
- Time elapsed until nodes achieve network connection (includes synchronization and RPL network joining time).
- Power consumption, related to the amount of traffic exchanged and the time the radio of nodes is active (either listening or transmitting).

### 6.1. Connection Success Rate

Table 7 shows the probability of connection success for each of the evaluated configurations during the 15 simulations. As can be seen, there are configurations where the joining node could not achieve the desired connection within one hour of simulation time. This limit is set as the maximum time that is acceptable for the connection of a new node to the network. If this limit is exceeded, it is considered as failure to connect. It is important to note that this value of time in simulations where the nodes have not achieved connection is discarded in order to avoid artificially alter the maximum values with arbitrary chosen values, so the results shown for the rest of KPIs must be always considered in agreement with the connection success rates.

### 6.2. Maximum Time for Achieving Network Connection

**Figure 15:**
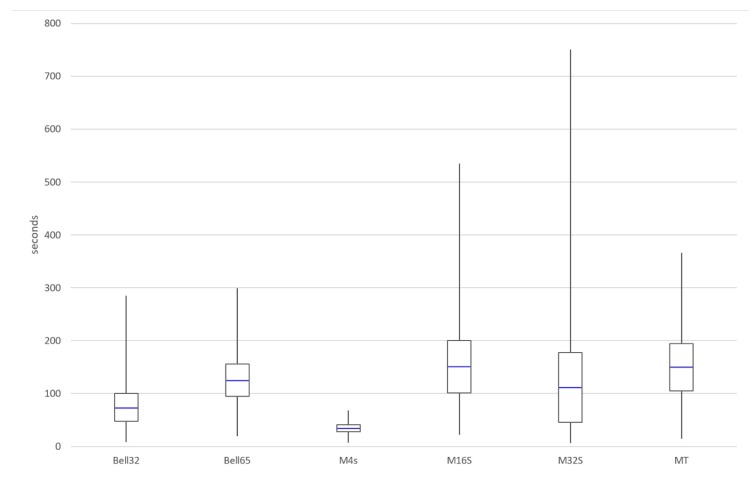
Statistical comparison of the different simulated configurations.

Figure 15 shows the maximum value, the minimum value, the average value, and the coverage for three standard deviation of the time needed by a new node to join effectively the RPL network so it can start transmitting data. All of the statistical measurements have been obtained from the average of the 15 simulations for each of the configurations.

The first thing to consider when looking at these results involves the M4 configuration, which shows the best values in connection time by far in comparison with the rest of configurations, but only because simulation where nodes did not connect were not computed in the calculation. When the node connects, it connects fast, but on 20% of the occasions, the node will never connect which is probably unacceptable in most situations, so this M4s configuration is likely to be discarded in favour of other solutions. M32 also has a better average than Bell-65, but with a greater dispersion of connection times, reaching very high maximum values without guaranteed connection.

The results show that the configurations Bell-32 and Bell-65, which are implementations of the proposed mechanism, achieve the best results in terms of average and dispersion in connection time while being the only configurations that are able to achieve 100% connection success.

### 6.3. Power Consumption

The main objective of Bell-X mechanism is to speed up the connection process while the energy consumption that is related to the advertising traffic is reduced. Equation (2) is proposed to be able to compare the different simulated configuration in terms of energy. In the numerator, the number of beacons transmitted during a bell cycle is obtained, while the total time of a bell cycle is calculated in the denominator. This equation allows for the average EB per second to be calculated using all of the configuration parameters detailed in Table 2.

**Figure 16:**
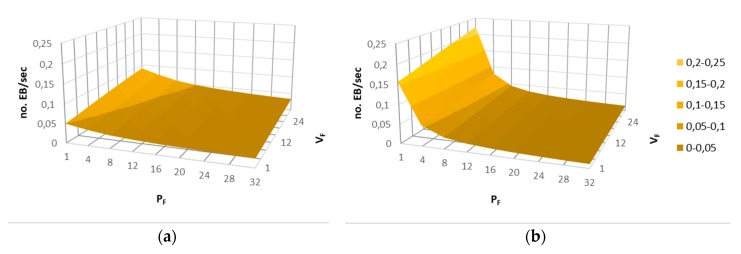
Number of beacons per second for different Bell-X configurations; (a) Fixed parameters SF = 4, Imin = 4, D = 4; (b) Fixed parameters S_F_ = 4, I_min_ = 1, D = 8.

Figure 16 shows the values obtained from the proposed model for two different configurations in which the V_F_ and P_F_ configuration parameters are modified. It can be seen how when the value of P_F_ increases, the number of beacons transmitted decreases, because the peak zone of the bell is longer. Nevertheless, if the value of V_F_ is greater, the bell spends more time at its minimum value, so a greater number of beacons is sent. Using this model, a user could choose an energy optimized configuration, which ensures that nodes connect faster in valley zones than in other configurations.

(2) $$EB/sec=\frac{V_{F}+2\cdot \left(D-1\right)\cdot S_{F}+P_{F}}{V_{F}\cdot I_{min}+2\cdot S_{F}\cdot \sum_{i=1}^{D-1}I_{min}\cdot 2^{i}+P_{F}\cdot I_{min}\cdot 2^{D}}$$

**Figure 17:**
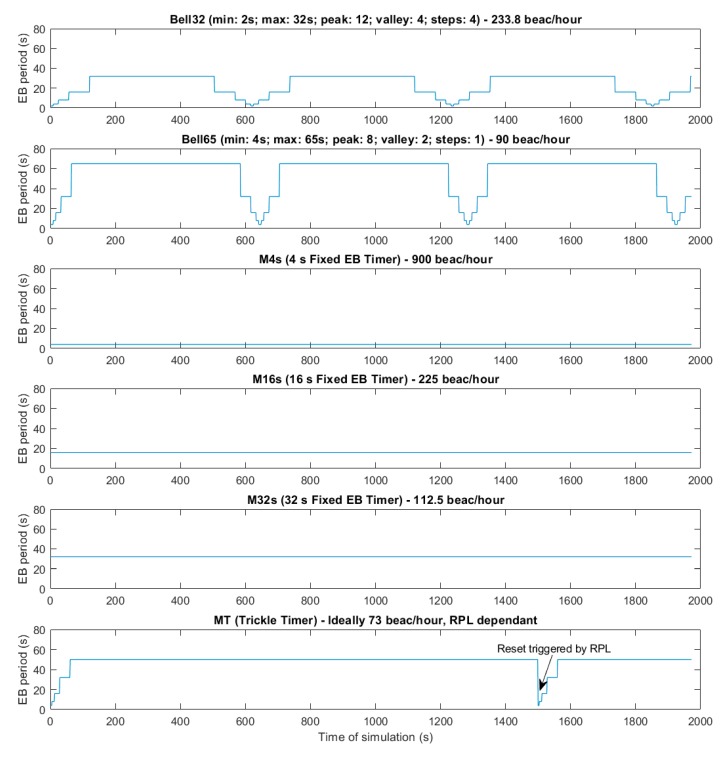
Comparison of the different simulated configurations in beacons per hour.

Figure 17 shows the quantity of EB messages exchanged for each configuration in order to extract value information regarding the impact of the proposed solution in power consumption. In this case, the Bell-65 achieves the lowest number of beacons/hour, only bested by the Trickle Timer. Nevertheless, the Trickle Timer configuration is dependent on the RPL layer behaviour. This means that the result shown is for the most optimistic case in which the timer is set by the RPL layer just rises to its maximum value of 50 s and stays there. In real conditions, the network condition will change over time, frequently restarting this timer and therefore increasing the number of beacons sent. This means that Bell-65 is the most energy efficient configuration tested regardless of the network condition.

**Figure 18:**
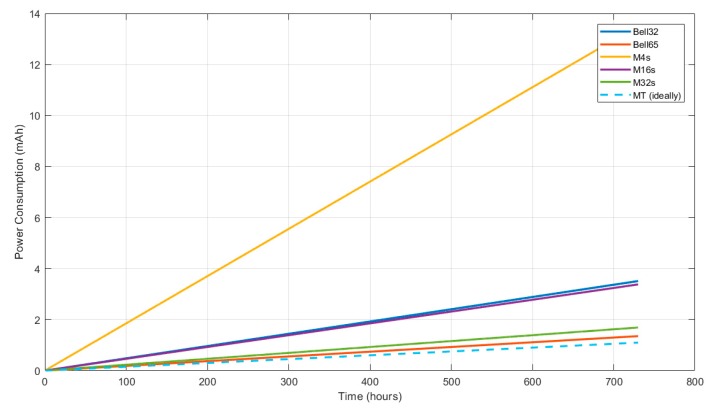
Consumption trend analysis during a month of operation for each configuration.

The effect of the different configurations on the evolution of power consumption, due to beaconing, for a node in the network, also taking into account the scanning phase (this is, the behaviour since the node is powered on), is shown in Figure 18. As can be seen, for higher values of ‘X’ in the Bell-X configuration (Bell-65), the energy used is rising slower than for the other configurations, leading to a longer node lifetime. Nevertheless, these results must be analysed while taking the possible connection time requirements and connection success rate into account.

## 7. Conclusions and Future Works

At the initial part of the article, the difficulties that are derived from using a MAC layer that requires synchronization, combined with a routing protocol that supports meshed networks, such as RPL, has been introduced. The proposal of using TSCH+RPL protocols to be used in industrial monitoring scenarios addresses the traditional problems wireless communications show in these types of environments, but still supposes a challenge to improve reliability during network joining time.

This work proposes a dynamic, tuneable mechanism, Bell-X, which allows for an adaptive beaconing method to be implemented, which can be configured to reduce power consumption and improve connection time and connection success rate to solve this challenge. This mechanism has been implemented and simulated, testing its results against the other popular solutions, as proposed in RFCs or WSN OS implementations.

**Figure 19:**
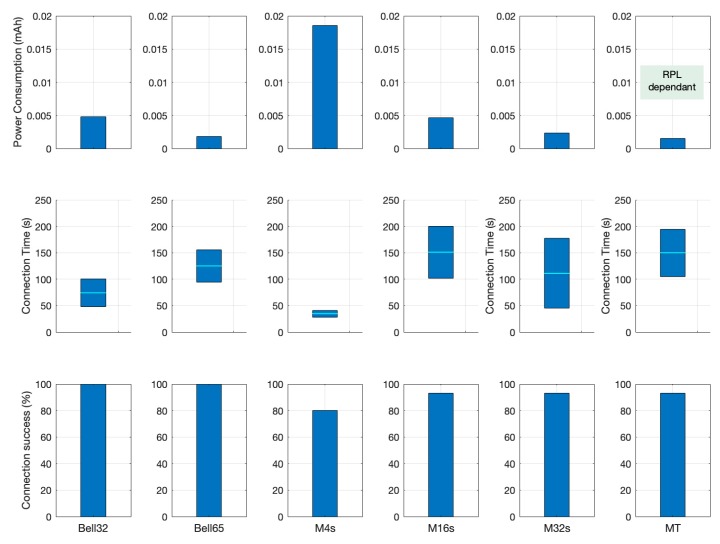
Key Performance Index (KPI) Comparison for tested configurations.

The described mechanism can be configured to respond to different requirements, but focusing on the results that were obtained for simulations and comparisons; it shows better overall results, especially for the Bell-65 configuration, looking for a compromise between success and time to connect and power consumption. Figure 19 illustrates the difference in KPIs achieved by the different mechanisms tested and helps to identify the best option depending on the application and use case necessities.

Bell-X mechanism is designed to be tuneable from the start, which means it is parameterized so that it could be used in combination with network and node state information, changing the shape of the bell. For instance, to enhance power savings, when batteries are low or the number of neighbours is high enough to transmit enough beacons, or even increase beaconing when the node is mains powered and adopts a role of time parents and relaying node to improve the connectivity. The configuration possibilities introduced by design in this solution open the way to future experiments and implementations, adapting to a wide range of use case and applications.

## Figures and Tables

**Table 1:** Success rate for beacon request simulations.

	Request RX by Node1	Requests TX	Time Elapsed before Synchronizing	Success Rate
Node2	15	1227	1:42:17.147	1.2%
Node3	10	1223	1:42:17.347	0.8%
Node4	18	1030	1:26:16.197	1.7%
Node5	22	1377	1:55:20.832	1.6%
Node6	13	873	1:13:14.367	1.5%
Total	78	5730		1.4%

**Table 2:** Configuration parameters for the proposed dynamic method.

Parameter	Description
Interval min (Imin)	This parameter corresponds to the minimum value of the beacon period, which coincides with the bell valley area.
Doublings (D)	It is the parameter that indicates how many times the timer is doubled before reaching its maximum value.
Interval max (Imax)	This parameter corresponds to the maximum value of the beacon period. This is a non-configurable parameter and corresponds to the result of doubling the value of the Interval min as many times as the Doublings parameter indicates which equation is $(I_{max}=I_{min}\cdot 2^{doublings}$).
$\mathrm{Valley}\;\mathrm{Factor}\;(V_{F})$	This value allows configuring the time that a node remains with the minimum beacon period. The duration of the timer in the valley zone (Valley Period) can be obtained by multiplying the value of the EB transmission period in that zone by the valley factor. In this way as many beacons can be sent as indicated in this parameter during the valley area.
$\mathrm{Step}\;\mathrm{Factor}\;(S_{F})$	This value allows configuring the time that a node remains in each of the steps between the minimum and the maximum beacon period. The duration of the timer in each of the step zones (Step Period) can be obtained by multiplying the value of the EB transmission period in that zone by the step factor. In this way as many beacons can be sent as indicated in this parameter during each of the step periods.
$\mathrm{Peak}\;\mathrm{Factor}\;(P_{F})$	This value allows configuring the time that a node remains with the maximum beacon period. The duration of the timer in the peak zone (Peak Period) can be obtained by multiplying the value of the EB transmission period in that zone by the value of the peak factor. In this way as many beacons can be sent as indicated in this parameter during the peak area.

**Table 3:** Simulation configuration.

Physical Parameters	
Distance Between Neighbours	40 m
Number of neighbours for central nodes (node 11)	4
Number of neighbours for nodes in the edges	3
Number of neighbours for nodes in the corners	2
Seeds per simulation	15
Minimum time before node 11 restart	20 min

**Table 4:** Protocol stack configuration.

	Parameters	Values
Global Parameters		
TSCH	Scan Duration	1 s
	Timeslot duration	10 ms
	TSCH Slotframe Schedule: Modified Orchestra Schedule [27] with multiple slotframes, each one for a particular traffic plane.	EBs: 101 slots in length with only two enabled (TX and RX) RPL: 31 slots in length with only one enabled (Shared)
	Number of Channels	4 channels
	Number of Scan Channels	4 channels
RPL	Trickle Timer Interval Min	4 s
	Trickle Timer Interval Doublings	8
	Trickle Timer Redundancy Constant	10
	DIS Interval	60 s

**Table 5:** Configuration parameters for the Bell-32 and Bell-65 configurations.

Parameters	Bell-32	Bell-65
Interval min	2 s	4 s
Doublings	4	4
Valley Period	4	2
Step Period	4	1
Peak Period	12	8

**Table 6:** Power consumption for the different slot types.

Description	Time TX Mode (ms)	Current Consumption (TX) (mA)	Time RX Mode (ms)	Current Consumption (RX) (mA)	Power Consumption (mAs)
Broadcast TX slot power consumption	4.256	17.4	-	19.7	0.0740544
Unicast TX slot power consumption	4.256		2.4 (ACK)		0.1213344
Broadcast RX slot power consumption	-		5.452		0.1074044
Unicast RX slot power consumption	2.4 (ACK)		5.452		0.1491644
RX slot without packet reception	-		2.2		0.04334
Scan phase	-		10		0.197

**Table 7:** Probability of synchronization/connection success.

	Synchronization Success	Connection Success
Bell-32	100%	100%
Bell-65	100%	100%
M4s	100%	80%
M16s	100%	93%
M32s	100%	93%
Trickle Timer	100%	93%
